# Molecular Biological Verification of the Healing Effect of Biphasic Microcurrent Electrical Stimulation in Model Rats of Skin Abrasion

**DOI:** 10.1155/2024/4549761

**Published:** 2024-09-16

**Authors:** Akira Sakaguchi, Yuzuru Sakaue, Shuhei Haraguchi, Daisuke Hasegawa, Rui Tsukagoshi, Kotaro Kawaguchi, Hideyuki Yamamoto

**Affiliations:** ^1^ Hyogo Medical University School of Rehabilitation Department of Physical Therapy, Kobe, Japan; ^2^ JCHO Osaka Hospital, Osaka, Japan; ^3^ PLAST Co., Ltd., Hyogo, Japan; ^4^ Medical Corporation Ikeikai Ando Surgery Orthopedics Clinic, Osaka, Japan

## Abstract

In this study, we investigated the effect of biphasic microcurrent electrical stimulation (b-MES) on the epidermal healing process using a rat model of skin abrasion. We analyzed the expression levels of growth factors [fibroblast growth factor 2 (FGF2) and epidermal growth factor (EGF)] and keratin subtypes (K10) in both the b-MES and control groups at different time points after wounding. The b-MES group showed a significantly accelerated healing process of the epithelial tissue, resulting in more consistent healing as compared to the control group. A molecular biological analysis showed that the FGF2 mRNA expression level on Day 2 after wounding was significantly higher in the b-MES group, whereas the EGF mRNA expression level on Days 1, 2, and 4 after wounding was significantly lower in the b-MES group. Additionally, the K10 mRNA expression level on Days 1 and 2 after wounding was significantly higher in the b-MES group. Our study findings suggest that b-MES facilitates wound healing by regulating the growth factors. However, the precise mechanisms underlying these effects remain to be fully elucidated. Further research is needed to fully understand the therapeutic potential of b-MES and its applications in clinical setting. Clinically, m-MES requires shunting due to residual electrical charge at the application site. However, b-MES alternates polarity, leaving no charge at the site of application. Therefore, b-MES also has the advantage of being safer and allowing treatment for longer periods of time.

## 1. Introduction

**Figure 1:**
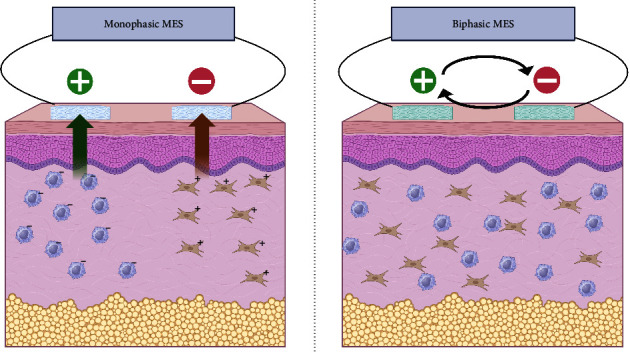
Differences between monophasic MES and biphasic MES. The wound-healing accelerating effect of MES was considered to be based on the electrical migration of various cells. For example, negatively charged macrophages are attracted to positive electrodes, while positively charged fibroblasts are attracted to negative electrodes. However, b-MES does not promote electrical migration by polarity switching. Therefore, the mechanism of action of MES may not be electrical migration.

Microcurrent electrical stimulation (MES) is an electrical stimulation therapy using subsensory currents of <1000 *μ*A [1, 2]. MES has been used to promote wound healing, such as pressure ulcers and venous ulcers, and has been widely reported [2, 3]. The mechanism of wound healing acceleration by electric stimuli, is thought the migration of macrophages and fibroblasts are induced to the wound area, in case of monophasic MES (m-MES) which is commonly used by physiotherapist [3, 4]. In acute stage, negatively charged macrophages attract to the anode side placed near the wound. Then, recovery period following the acute stage, positively charged fibroblasts attract to the cathode side (Figure 1).

Furthermore, MES is often used for recovering from delayed onset muscle soreness [5–7], promote healing of acute musculoskeletal disorders [8, 9], and reducing pain and swelling after surgery. Therefore, it is thought to have some improvement effect on acute tissue damage [10].

Interestingly, it is reported that the electric stimuli whether it is monophasic or biphasic, induce mRNA expression and protein synthesis of growth factors, recently [11–14]. Therefore, it has been suggested that the electric stimuli not only induce cell migration, but also promoting cell division through induction of growth factor's mRNA expression and protein synthesis to accelerate wound healing. Experimental models for studying soft tissue healing include tendon [8, 15], muscle [9, 16], and skin wound models. Skin wound models include full-thickness defect models [17, 18] and partial defect models [19–21], and the method chosen is appropriate for the purpose of the study. The full-thickness defect model damages the epidermis, dermis, and subcutaneous tissue, and therefore many factors are involved in the healing process. In molecular biological analysis, it is difficult to determine in which layer of the wound repair process growth factors and various substances are expressed. Therefore, in this study we used an abrasion model in which only the epidermis is injured to investigate the effect of b-MES on epidermal healing.

In abrasion models, healing is thought to be accelerated by increased production of epidermal growth factor (EGF), fibroblast growth factor (FGF), and the stratum corneum. In this study, we investigated whether b-MES affects the mRNA expression of EGF, and FGF2 in a rat skin ablation model.

## 2. Subjects and Methods

### 2.1. Subjects

Thirty-two 6-week-old male Wistar rats (Crlj/WI) were used. Two abrasion wounds were made on the ventral skin of 24 rats, and b-MES was performed once daily immediately after the abrasion wounds were made to form the b-MES group. Tissues were collected from four groups (*n* = 6/group) on each of the following days: Day 1, Day 2, Day 3, and Day 4 after the creation of the abrasion wounds.

Two samples were taken from each animal, one for histological analysis and the other for molecular biological analysis.

On the other hand, three abrasion wounds were made on the lateral skin of the ventral side of 8 rats, which served as a control group without b-MES. Tissues were collected from four groups (*n* = 2/group) on each of the following days: Day 1, Day 2, Day 3, and Day 4 after the creation of the abrasion wounds.

**Figure 2:**
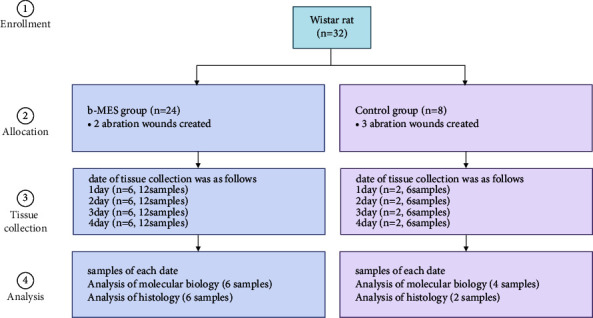
Protocol of study. Thirty-two Wistar rats were divided into 24 for b-MES and 8 for control rats. Rats in the MES group had two abrasion wounds on the lateral skin of the ventral side, and rats in the control group had three abrasion wounds on the lateral skin of the ventral side. In each group, rats were divided into groups for tissue collection on Days 1, 2, 3, and 4. In the MES group, 12 samples were obtained on each collection day. Six samples each were used for histological and molecular biological analysis. In the control group, 6 samples were obtained, 2 for histological analysis, and 4 for molecular biological analysis.

Three samples were taken from each animal, one for histological analysis and the others for molecular biological analysis (Figure 2).

The rats were housed in pairs within a single cage, allowing them to move freely. The cages were maintained under a controlled 12 hour light/dark cycle, ensuring a regular alternation between light and darkness every 12 hours. Throughout the study, the rats had unrestricted access to water and food, which was provided ad libitum. This setup facilitated normal animal behavior and contributed to their overall welfare by providing a stable and humane environment.

During the experiment, the rats were free to move around the cage and had free access to water and food.

### 2.2. Creation of Abrasion Wounds

Rats were anesthetized with an intraperitoneal mixture of medetomidine hydrochloride (0.15 mg/kg), midazolam (2 mg/kg), and butorphanol tartrate (2.5 mg/kg). At first, a part of the ventral side hair of the anesthetized rats was shaved with electric clippers. For abrasion wounds, a piece of paper with an 8 mm diameter hole was placed on the skin and abraded inside the hole for 10 seconds, by a handy multi-tool router (Kin-Power Tool No. 28473, Proxxon) with a No.150 grindstone.

### 2.3. MES Intervention

The b-MES was performed for 30 minutes daily from the day of wound preparation until the day before tissue collection. Rats were anesthetized by intraperitoneal administration of medetomidine hydrochloride (0.15 mg/kg), midazolam (2 mg/kg), and butorphanol tartrate (2.5 mg/kg), and placed in the supine position. Electrodes were placed across the wound. Ultrasound gel (Ito Co., Ltd.) was applied to reduce electrical resistance to efficiency of current flow. A portable electrical stimulator (Ito Co., Ltd., Trio 300) with biphasic polarity, square waveform, 0.3 Hz frequency, and 500 *μ*A intensity was used.

### 2.4. Histological Analysis

Skin samples were obtained from euthanized rats, and skin tissue was obtained using an 8 mm diameter biopsy punches (Kai Industries Co., ltd.) and stored in a −80°C freezer. Tissues were thinned to 10 *μ*m using a cryostat (Carl Zeiss Co., Ltd. HYRAX C50) and stained with hematoxylin and eosin (HE). Tissue sections were then examined using a light microscope (OLIMPUS Co., Ltd., CX-41). All tissue images were photographed with a digital camera (NIKON Co., Ltd., D5100).

### 2.5. Tissue Collection and RNA Isolation and Purification

For tissue collection, rats were euthanized, and skin tissue was collected using an 8 mm diameter biopsy punches (KAI Industries Co., ltd.) and stored in a −80°C freezer. Immediately prior to total RNA purification, samples were crushed in a cryopress (Microtech Co., Ltd.) and dissolved in TRIZOL solution (Ambion). Total RNA purification followed the protocol supplied with TRIZOL.

Skin tissue is one of the most difficult tissues to purify for total RNA, and the protocol provided with TRIZOL does not ensure the purity required for mRNA quantification, so the following step was added. Total RNA dissolved in 200 *μ*l of deionized distilled water was mixed with an equal volume of TE-saturated phenol (pH 8.0, Nippon Gene Co., Ltd.) by vortexing and centrifuged at room temperature and 12000 rpm for 3 min to collect the supernatant three times, and an equal volume of chloroform (special grade, Nakalai Tesque, Inc.) was added. The supernatant was collected by centrifugation at 12000 rpm for 3 min at room temperature after vortexing with an equal volume of chloroform (special grade, Nakalai Tesque, Inc.). 20 *μ*l of sodium acetate (3 M. pH 7.4, Nakalai Tesque, Inc.) was added to the total RNA and the volume was adjusted to 200 *μ*l by the addition of deionized distilled water. 900 *μ*l of 100% ethanol (special grade, Wako Pure Chemical Industries, Ltd.) was added and cooled in a freezer at −20°C for 15 min. After centrifugation at 4°C, 12,000 rpm for 15 min, the supernatant was discarded, 800 *μ*l of 70% ethanol was added, centrifuged at 4°C, 12,000 rpm for 3 min, the supernatant was discarded and air dried. In addition, the following steps were performed to eliminate the genomic DNA fragments contaminated in the total RNA completely. The air-dried total RNA was dissolved in 176 *μ*l of deionized distilled water, 2 *μ*l of DNase (Nippon Gene Co., Ltd.), 20 µl of 10×DNase I buffer (Nippon Gene Co., Ltd.) and 2 *μ*l of RNase inhibitor (Nippon Gene Co., Ltd.) were added and incubated at 37°C for 30 minutes. Phenol and chloroform treatments were then performed twice each, followed by ethanol precipitation. Additionally, because the amount of total RNA recovered from the skin was expected to be very low, 2 *μ*g of glycogen (Roche Diagnostic K.K.) was added to all samples before performing the final ethanol precipitation of purification protocol to increase recovery efficiency. The final total RNA precipitate was dissolved in an appropriate volume of deionized distilled water and quantified using an ultra-trace spectrophotometer (NanoDrop 1000, Thermo Fisher Scientific Inc).

Keratin subtype K1 and K10 mRNA, fibroblast growth factor (FGF) and epidermal growth factor (EGF) mRNA expression levels were measured by real-time polymerase chain reaction (RT-PCR). The all-gene specific primers were designed by Oligo 7 Primer Analysis Software (Molecular Biology Insights Inc.) (Table 1).

For mRNA quantification of each gene, RNA direct RT-PCR Master Mix (Toyobo Life Science Co., Ltd.) was used, and the attached protocol was followed. The first strand DNA synthesis was performed at 61°C for 20 min. Real-Time PCR was performed as a following conditions; the initial denaturation was performed at 98°C for 2 min, followed by 45 cycles at 98°C for 1 sec, 67°C for 15 sec, and 74°C for 35 sec. One hundred nanogram of total RNA were used for each reaction tube. The expression level of each gene obtained by the RT-PCR was normalized by the expression level of Glyceraldehyde-3-phosphate dehydrogenase (GAPDH), which is known as a internal control.

### 2.6. Statistical Analysis

The mRNA expression levels of the b-MES group and the control group were compared between the two groups using the Mann–Whitney *U* test. In all analyses, statistical analyses were carried out using R (ver. 2.8.1), and an effect was considered statistically significant if its associated *p* value was smaller than 0.05.

### 2.7. Ethics

This experiment was conducted under the approval of the Animal Experimentation Committee of Hyogo University of Medical Sciences (Approval No. 2017-11).

## 3. Results

### 3.1. Histological Analysis

On Day 1, epithelial tissue damage was confirmed in both the b-MES and control groups; on Day 2, the control group showed epithelial tissue damage as in Day 1, whereas the b-MES group showed that the epithelial tissue had begun to heal.

**Figure 3:**
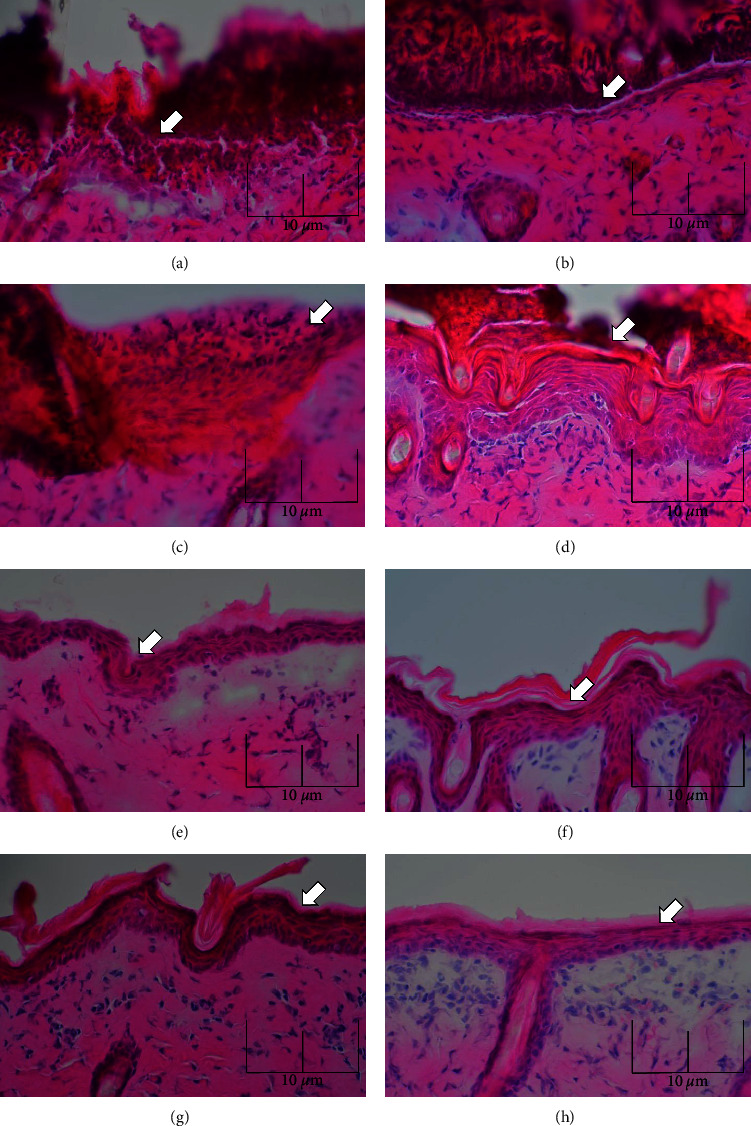
Histological observations. The left side is the control group and the right side is the MES group. From the third day of abrasion wound creation, epithelialization progressed more in the MES group than in the control group, confirming the epithelialization-promoting effect of b-MES. (a, b) Day 1. In both groups, epithelial tissue damage was observed. (c, d) Day 2. The MES group showed epithelialization. (e, f) Day 3. In the MES group, a uniform epithelial layer was observed. (g, h) Day 4. Epithelialization was more advanced in the MES group.

On Day 3 and Day 4, healing of the epithelial tissue was confirmed in both the b-MES and control groups, but the b-MES group showed more uniform healing of the epithelial tissue than the control group (Figure 3).

**Figure 4:**
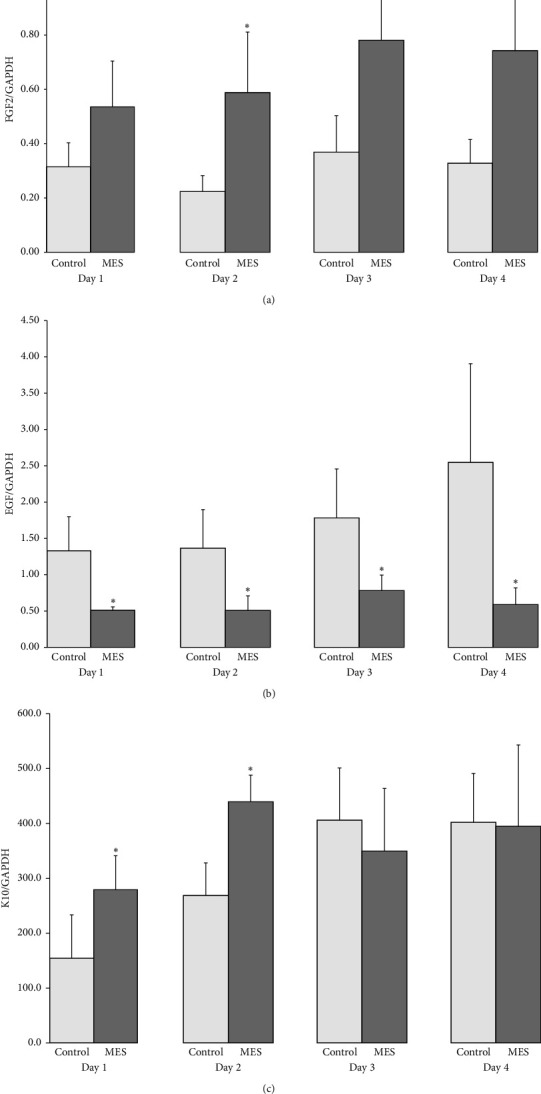
Gene expression of FGF2, EGF, and K10 was significantly different between the MES group and the control group at different time points. (a) FGF2 mRNA expression level was significantly increased after Day 2. (b) EGF mRNA expression level was significantly decreased on all days. (c) K10 mRNA expression level was significantly increased on Day 1 and Day 2. ^∗^*p* < 0.05.

mRNA expression analysis by molecular biological method. The expression levels of growth factors and keratins were compared between the b-MES and control groups on each day. In all cases, the control and b-MES groups are listed in this order (Figure 4).

### 3.2. FGF2 mRNA and EGF RNA

FGF2 was present on Day 1 (control: 0.32 ± 0.09, b-MES 0.54 ± 0.17), Day 2 (0.23 ± 0.06, 0.59 ± 0.22), Day 3 (0.37 ± 0.13, 0.78 ± 0.32), and Day 4 (0.33 ± 0.09, 0.74 ± 0.19). EGF was present on Day 1(1.33 ± 0.47, 0.51 ± 0.05), Day 2(1.36 ± 0.53, 0.50 ± 0.20), Day 3(1.79 ± 0.67, 0.78 ± 0.21), and Day 4(2.56 ± 1.35, 0.58 ± 0.23).

According to our data, it is clear that the expression level of FGF2 mRNA of b-MES group was significantly higher than control group for 2 to 4 days after wounding. On the other hand, the expression level of EGF mRNA was lower in the b-MES group than control group during the entire observation period (Figures 4(a), 4(b)).

### 3.3. K10 mRNA

K10 mRNA expression level was significantly higher in the b-MES group than in the control group on Day 1 after wound preparation: 154.0 ± 79.3, 278.8 ± 62.5 on Day 1, 269.0 ± 59.0, 439.4 ± 49.0 on Day 2, 406.5 ± 94.0, 349.9 ± 113.4 on Day 3, and 402.2 ± 88.7, 395.1 ± 147.3 on Day 4.

## 4. Discussion

Histological analysis confirmed that b-MES accelerated the epithelialization of superficial dermal abrasions. These results support previous reports on the effect of MES on wound healing. We found that the expression level of FGF2 mRNA of b-MES group was significantly higher than control group, but the EGF mRNA level was lower in the b-MES group. In case of K10 which is the stratum spinosum and stratum granulosum marker gene, the mRNA expression level was higher in b-MES group Days 1 and 2 after wounding.

In this study, the expression level of FGF2 mRNA was significantly increased in the MES group on Days 2, 3, and 4 after wounding compared with the control group. On the other hand, the expression level of EGF mRNA was significantly decreased during the entire observation period.

It is known that FGF2 is involved in epithelial tissue healing, including fibroblast migration, angiogenesis, granulation tissue growth, matrix remodeling and re-epithelialization. Previous studies have reported the effect of the artificial addition of FGF2 on wound healing and its activation in relation to wound healing [22–24]. Wound healing studies using FGF2 knockout mice reported that wound size, crust thickness, epithelialization, and collagen deposition were delayed compared to normal mice [25]. In the case of EGF, it has been reported that forced addition promotes wound healing, and this is thought to be because EGF promotes cell differentiation of keratinocytes, fibroblasts, and vascular endothelial cells [26–29]. Thus, both FGF2 and EGF have the effect of promoting wound healing, but it is necessary to clarify whether it is desirable for the expression levels of the two factors to increase at the same period during wound healing. Interestingly, previous investigation showed that the effects of multiple growth factors addition, wound healing tended to be promoted by FGF2 alone, but not in combination with EGF, suggesting that EGF may inhibit the FGF2 action [30]. Therefore, our data support the previous studies and suggest that FGF2 expression needs to be increased and EGF expression needs to be suppressed to promote wound healing.

The expression level of K10 keratin mRNA showed a significant increase in the b-MES group compared to the control group on Days 1 and 2 after wounding. The type of keratin expressed in epidermal cells varies depending on the cell type and the degree of differentiation. In normal skin, K5/K14 is expressed in the basal layer and K1/K10 in the stratum spinosum and stratum granulosum, whereas K10 keratin in wounded skin is reported to decrease immediately after wounding and increase as the wound closes. Patel et al. reported a decrease in K10 at the wound edge on Day 2 after wound creation and an increase with wound closure in a full-thickness defect model [31]. Koizumi et al. reported that during the healing process, K10 is expressed slightly later during cell differentiation [32].

The results of this study showed that b-MES significantly increase the expression level of K10 mRNA compared to the control group on Day 1, suggests that b-MES promotes epithelialization through enhanced cell differentiation in the stratum spinosum and stratum granulosum.

Previous studies have reported that electrical stimulation promotes wound healing by inducing cell migration [3, 33], enhancing protein and DNA synthesis, and increasing the expression of collagen and elastin [11]. Additionally, various studies have shown that electrical stimulation promotes cytokine secretion [34] and increases levels of growth factors involved in wound healing, such as FGF2 [35], EGF [36], and VEGF [37].

However, most of these studies used m-MES, which applies electrical stimulation under unidirectional conditions. In contrast, we investigated the wound healing effects of b-MES, which appears to have a lower capacity for inducing cell migration compared to m-MES. Our results suggest that b-MES promotes wound healing by inducing FGF2 expression, suppressing EGF expression, and promoting cell differentiation in the spinous and granular layers, as indicated by K10 expression analysis.

Furthermore, conventional wound healing studies in vivo commonly use whole-layer defect models, which do not distinguish the contributions of epithelial and dermal tissues to the upregulation of growth factors and other substances. In this study, we employed an abrasion model and confirmed that the upregulation of FGF2 occurs specifically in epithelial tissue.

The mechanism underlying the effects of b-MES remains unclear. However, it has been hypothesized that electrical actions, such as increased ATP production [38], which promotes membrane ion translocation and cAMP activation [39], may be involved. Clinically, m-MES requires shunting due to residual charge at the application site, whereas b-MES alternates polarity and thus leaves no residual charge, making it safer and more suitable for prolonged treatment.

Recent advances in nanogenerator technology have led to the development of therapeutic patches that generate continuous biphasic pulsed currents, which have been reported to promote wound healing [40, 41]. The observation that b-MES promotes the healing of abrasions in this study is a valuable contribution to establishing more advanced treatment protocols.

## 5. Conclusion

The b-MES in an ablation rat model may promote epithelialization by promoting cell differentiation in the stratum spinosum and/or stratum granulosum through induction of FGF2 mRNA expression and suppression of EGF mRNA expression. The molecular mechanism of wound healing by b-MES is not clarified yet; further studies will increase the benefit to patients by expanding its use in clinical practice as a safe and easy-to-use modality.

## Figures and Tables

**Table 1:** Primer details of growth factors and K10.

Gene	S/A	Primer sequence	Accession no.
GAPDH	Sense	5′-TTCCTTTCGCAAAACAAGTTCACC-3′	AF106860
	Antisense	5′-TAGGAGCTTGACTTACAGAAGAATCGTT-3′	

FGF2	Sense	5′-ATTTCCAAAACCTGACCCGAT-3′	NM_019305
	Antisense	5′-CCTGCCTTTTAACACAACGACCA-3′	

EGF	Sense	5′-TGTCACAGCGAGAAATCAGTVACC-3′	NM_012842
	Antisense	5′-GCTGTTTTAATACCTGACACCCGTA-3′	

K10	Sense	5′-CCTGCAAATAACCCTCAATTGCTT-3′	NM_001008804
	Antisense	5′-ATCACTCTGGTTTTATTAGTATTGCTCCC-3′	

## Data Availability

The data that support the findings of this study are available from the corresponding author upon reasonable request.
